# Supplementary material for: MetaQTL: a package of new computational methods for the meta-analysis of QTL mapping experiments
Source: BMC Bioinformatics. 2007 Feb 8;8:49. doi: 10.1186/1471-2105-8-49 (PMC1808479; doi:10.1186/1471-2105-8-49)
Supplement: Additional File 7 — MetaQTL Package : jar file and tutorial. This Zip archive contains both the MetaQTL JAR file and the files of the tutorial. [file 1471-2105-8-49-S7.zip › org.inra.metaqtl/doc/overview-summary.html]

Overview


|  |  |  |
| --- | --- | --- |
| **Overview** \| Package \| Class \| Use \| **Tree** \| **Deprecated** \| **Index** \| **Help** | |  |
| PREV   NEXT | **FRAMES**    **NO FRAMES**     **All Classes** |  |


---

| **Packages** | |
| --- | --- |
| **org.metaqtl** |  |
| **org.metaqtl.adapter** |  |
| **org.metaqtl.algo** |  |
| **org.metaqtl.bio** |  |
| **org.metaqtl.bio.entity** |  |
| **org.metaqtl.bio.entity.adapter** |  |
| **org.metaqtl.bio.entity.bean** |  |
| **org.metaqtl.bio.entity.factory** |  |
| **org.metaqtl.bio.util** |  |
| **org.metaqtl.factory** |  |
| **org.metaqtl.graph** |  |
| **org.metaqtl.main** |  |
| **org.metaqtl.numrec** |  |
| **org.metaqtl.util** |  |

---


|  |  |  |
| --- | --- | --- |
| **Overview** \| Package \| Class \| Use \| **Tree** \| **Deprecated** \| **Index** \| **Help** | |  |
| PREV   NEXT | **FRAMES**    **NO FRAMES**     **All Classes** |  |


---
